# Menstruation Dysregulation and Endometriosis Development

**DOI:** 10.3389/frph.2021.756704

**Published:** 2021-10-13

**Authors:** Kevin K. W. Kuan, Douglas A. Gibson, Lucy H. R. Whitaker, Andrew W. Horne

**Affiliations:** ^1^Medical School, University of Edinburgh, Edinburgh, United Kingdom; ^2^Centre for Inflammation Research, University of Edinburgh, Edinburgh, United Kingdom; ^3^Medical Research Council (MRC) Centre for Reproductive Health, University of Edinburgh, Edinburgh, United Kingdom

**Keywords:** endometriosis, menstruation, pathogenesis, inflammation, matrix metalloproteinase (MMP), angiogenesis, apoptosis, abnormal uterine bleeding (AUB)

## Abstract

Endometriosis is a common gynecological condition characterized by the growth of endometrial-like tissue outside of the uterus which may cause symptoms such as chronic pelvic pain or subfertility. Several surgical and medical therapies are available to manage symptoms, but a cure has yet to be determined which can be attributed to the incomplete understanding of disease pathogenesis. Sampson's theory of retrograde menstruation is a widely accepted theory describing how shed endometrial tissue can enter the peritoneal cavity, but other factors are likely at play to facilitate the establishment of endometriosis lesions. This review summarizes literature that has explored how dysregulation of menstruation can contribute to the pathogenesis of endometriosis such as dysregulation of inflammatory mediators, aberrant endometrial matrix metalloproteinase expression, hypoxic stress, and reduced apoptosis. Overall, many of these factors have overlapping pathways which can prolong the survival of shed endometrial debris, increase tissue migration, and facilitate implantation of endometrial tissue at ectopic sites. Moreover, some of these changes are also implicated in abnormal uterine bleeding and endometrial diseases. More research is needed to better understand the underlying mechanisms driving dysregulation of menstruation in endometriosis specifically and identifying specific pathways could introduce new treatment targets. Analyzing menstrual fluid from women with endometriosis for inflammatory markers and other biomarkers may also be beneficial for earlier diagnosis and disease staging.

## Introduction

Endometriosis is a chronic inflammatory condition characterized by the growth of endometrial-like tissue outside the uterus. Around 10% of reproductive-aged women are affected and symptoms may include chronic pelvic pain, dyspareunia, and subfertility which can impair the patient's quality of life and work productivity (1). Paradoxically, the severity of symptoms does not necessarily correlate with disease presentation and the lack of reliable diagnostic biomarkers contributes to the average diagnostic delay of 7 years from the onset of symptoms (2, 3). Depending on location and depth of tissue invasion, endometriosis can be classified as superficial (peritoneal), ovarian, or deep (infiltrating) endometriosis. If endometriosis is suspected, laparoscopic visualization remains the gold standard for diagnosis and lesions found may be excised or ablated for symptom control. Apart from surgery, medical treatments, such as analgesics, hormonal modulation/suppression with progestins, combined oral contraceptive pills, or Gonadotrophin-releasing hormone (GnRH) modulators, can be used for pain management. While symptoms may be reduced temporarily, a cure has sadly not been identified.

One of the main challenges for researchers is the uncertainty regarding the exact underlying mechanisms explaining the etiology and natural history of endometriosis. Sampson's theory of retrograde menstruation is the most widely accepted hypothesis describing how disruption to normal menstrual flow may result in endometriosis. Normally, the superficial (functional) endometrial layer is sloughed during menstruation (menses) to prepare the endometrium for the next menstrual cycle resulting in vaginal bleeding for an average of 5 days (4). In retrograde menstruation, shed tissue flows through the fallopian tubes, enters the pelvic cavity and adheres to tissue in the pelvic cavity leading to formation of ectopic endometriosis lesions.

This review focuses on the “theory of retrograde menstruation” as part of the Frontiers “Menstruation: Myths, Mechanisms, Models, and Malfunction” Special Issue. However, we acknowledge that retrograde menstruation is unable to explain all cases of endometriosis and other theories [e.g., stem cells, epithelial-mesenchymal transition (EMT), coelomic metaplasia, etc.] have also been proposed (5–7).

## Search Strategy

We applied a broad search strategy to the PubMed database using the terms “(endometriosis) AND [menstruation OR (menstrual cycle) OR menses]” for studies published from inception until July 2021 yielding 3,020 manuscripts. Studies not specific to the menstruation dysregulation themes or other narrative review articles were excluded. Reference lists of studies used in this review were searched for additional studies we judged as relevant. Ultimately, 62 studies were included in the review.

## Regulation of Menstruation

### Dysregulation of Inflammatory Mediators

Following ovulation, the corpus luteum produces progesterone which has anti-inflammatory effects to create a suitable environment for embryo implantation. In the absence of pregnancy, the corpus luteum regresses causing a rapid decline in progesterone levels increasing the activation of the NF-kB inflammatory pathway to prepare for menstruation (8, 9). Local endometrial secretion of inflammatory mediators from epithelial and stromal cells are normally upregulated during the secretory and menstrual phase in the presence of tissue necrosis to aid endometrial repair as part of physiologic menstruation (8).

Many studies have investigated the role of inflammatory regulators in the pathogenesis of endometriosis especially the production of interleukin-1, interleukin-8, tumor necrosis factor-a (TNF-a), monocyte chemotactic protein-1 (MCP-1), and macrophage migration inhibitory factor, and it is generally agreed that women with endometriosis display significantly higher cytokine mRNA expression and immunohistochemistry staining in eutopic/ectopic endometria and peritoneal tissue (10–12). Increased cytokine secretion from endometrial tissue and peritoneal fluid may act in an autocrine manner to promote angiogenesis and cellular proliferation in the endometrium prolonging the viability of shed endometrial cells for implantation, but the exact relationship remains unclear (10).

Apart from aberrant cytokine expression, their receptors are also dysregulated in endometriosis. For example, soluble IL-1 receptor II (ILR-II) is normally concomitantly upregulated as a “decoy receptor” inhibiting excess activation of IL-1. In endometriosis, researchers observed a downregulation of ILR-II immunostaining in eutopic endometrial tissue (13) and greater IL-1-induced MCP-1 secretion from endometrial epithelial cells *in vitro* (14). Interestingly, Akoum et al. observed increased ILR-II staining within epithelial cells suggesting that the release of ILR-II to the cell surface may be inhibited (13). TNF-a also has two primary receptors, TNF-RI and TNF-RII, with pro-inflammatory and anti-inflammatory actions, respectively. In endometriosis, decreased expression of anti-inflammatory TNF-RII within endometrial glandular cells favors pro-inflammatory activity and reduced apoptosis, the importance of which will be discussed later (15). The underlying causes for the downregulated receptors are uncertain and improved knowledge of these control pathways could introduce new treatment methods to reduce the exaggerated immune response.

Dysregulation of innate and adaptive immune mediators could also promote the development of endometriotic lesions. Studies found that antigen-presenting cells like dendritic cells (DC) and Foxp3+ regulatory T-cells were downregulated in endometriosis during the secretory and menstrual phases (16, 17). Although their exact role in menstruation is unclear, researchers suggest that they may activate a targeted immune response toward menstrual debris for clearance (16, 17). The function of endometrial macrophages also appears altered in endometriosis which can cause implications toward disease progression (18). Normally, macrophages phagocytose foreign substances but this activity can be suppressed by certain regulators. For example, mRNA expression of the scavenger receptor CD36 is decreased in peritoneal macrophages and may explain decreased phagocytosis in women with endometriosis contributing to the persistence of peritoneal cavity lesions (19). If endometriotic lesions bleed, this can cause peritoneal heme accumulation and increased heme oxygenase-1 (HO-1) expression in ectopic endometrial stromal cells and peritoneal macrophages, both suppressors of phagocytosis (20). Although evidence suggests that heme and HO-1 overload reduces phagocytosis of ectopic stromal cells, whether these factors could affect endometrial macrophage activity in a paracrine manner should be explored (20). Macrophage phenotype can also exhibit pro- and anti-inflammatory properties. A recent study sequenced RNA from eutopic endometrial macrophages from women with endometriosis which exhibited a significantly greater (*z*-score ≥ 2.00) pro-inflammatory phenotype (activation of NF-kB pathways and increased upstream TNF regulators) not observed in controls (21). Altogether, an inefficient clearance of shed menstrual fragments could prolong the survival of cells increasing the chance for implantation. Furthermore, the presence of uncleared debris and altered macrophage phenotype may further contribute to an inflammatory peritoneal environment promoting the establishment and persistence of endometriosis lesions (19–21).

Limited research has been done analyzing menstrual blood for inflammatory markers in endometriosis. One study found significantly higher myeloperoxidase (MPO) and N-acetyl-B-D-glucosaminidase (NAG) enzymes (*P* = 0.0117 and *P* = 0.039, respectively), both markers of leukocyte accumulation, in the menstrual blood of women with endometriosis compared to peripheral blood which was not observed in controls. However, when menstrual effluent NAG and MPO activity was compared between controls and endometriosis samples, there was no significant difference (22). Nonetheless, the significant difference in inflammatory markers found in the menstrual blood of endometriosis samples should not be undermined because they corroborate with earlier studies that suggest increased inflammatory activity in endometriosis (15). Recent evidence also found a distinct cytokine profile in menstrual blood vs. blood plasma in healthy donors demonstrating the importance of menstrual blood as a non-invasive source for profiling expression of mediators found in endometrial tissue (23). Therefore, future studies should consider utilizing menstrual blood for analyzing other inflammatory markers raised in endometriosis since it would best represent the inflammatory content of menstrual effluents during retrograde menstruation.

## Matrix Metalloproteinases

Matrix metalloproteinases (MMP) are a family of enzymes mainly localized in the functional layer of the endometrium and secreted from stromal fibroblasts and immune cells mediating endometrial breakdown and extracellular matrix remodeling during menstruation. Ovarian steroid hormones regulate MMP activity and endogenous antagonists known as tissue inhibitors of matrix metalloproteinases (TIMP) prevent overexpression (24). Due to their impact on endometrial structure, abnormal expression of some MMPs such as MMP-2 and MMP-9 are implicated in uterine pathologies such as heavy menstrual bleeding (HMB), fibroids and adenomyosis (25–27). In endometriosis, aberrant MMP/TIMP expression may cause excess endometrial tissue migration, endometrial invasion, and recruitment of angiogenic factors in ectopic lesions (28, 29). Furthermore, enhanced proteolytic activity may dislocate the basal layer of the endometrium increasing the amount of basalis cells in menstrual blood with stem cell characteristics that can differentiate into epithelial and stromal endometrial tissue supporting the stem cell theory (5, 30). However, not all subtypes are dysregulated in endometriosis, and several factors could influence MMP expression.

As mentioned earlier, inflammatory mediators have a multifaceted role and regulating MMP activity is no exception. In a study that treated uterine tissue containing both epithelial and stromal cells with cytokines upregulated in endometriosis, tissue derived from patients with endometriosis secreted more MMP-3 following IL-1 treatment (*P* < 0.01) in a dose-dependent manner which was not observed in controls. This showed that endometrial cells from women with endometriosis respond differently to cytokine-induced MMP secretion. However, treatment with TNF-a did not change MMP-3 secretion. Furthermore, MMP-1/2 and TIMP-1/2 levels were not significantly different to controls after cytokine stimulation suggesting that cytokine-specific pathways are present (31). Therefore, future *in vitro* analyses of MMP secretion against an array of cytokine treatments may be useful in identifying specific immune pathways. MMP-27 has also been found to localize near CD163+/CD206+ macrophages especially during the time of menstruation. This inflammatory and degenerative microenvironment can favor endometriosis progression by increasing tissue migration and promoting implantation (32).

Local MMP expression can vary greatly depending on the location of endometriotic lesions and growth patterns. One study analyzed MMP expression in colorectal endometriosis (one of the most aggressive forms of deep infiltrative endometriosis) and reported significantly greater MMP-2,−3, and−11, and lower TIMP-2 expression than endometrial cysts and peritoneal lesions (33). Ovarian endometriomas also had a different MMP profile with increased production of MMP-1,−2,−7, and−9 during the menstrual period (29, 33). Since ovarian steroid hormone secretion lacks the normal cyclic variation in the presence of endometriomas (34), this may explain the different MMP-2 expression compared to other types of endometriosis (29). *In vitro* studies suggest that increased MMP activity is related to disease invasiveness which may explain the different MMP profile in colorectal endometriosis (35, 36). Certain MMP levels in peritoneal fluid are positively correlated to advanced stages of disease according to revised AFS staging, making MMP a promising diagnostic biomarker and is being explored (37). The exact cause for the overall increased MMP-2 expression remains unknown, but it may be due to reduced MMP-2 gene methylation (38). For clarity, Table 1 summarizes the MMPs upregulated depending on endometriosis location and main study findings.

**Table 1:** Summary of literature analyzing MMP expression in endometriosis.

**References**	**Important MMP/TIMP subtype(s) identified**	**Location of endometriosis**	**Main finding(s)/conclusion(s)**
Sillem et al. (39)	TIMP-2	Unspecified	Increased TIMP-2 transcription may increase endometrial cell invasiveness
Mizumoto et al. (29)	MMP-1,−2,−7, -9 TIMP-1	Endometrioma	MMP primarily produced from stromal cells which may cause ECM destruction
Sillem et al. (31)	MMP-1,−2, -3 TIMP-1,−2	Unspecified	IL-1 induces significantly greater MMP-3 secretion from eutopic EM tissue not observed in controls
Chung et al. (35)	MMP-2 TIMP-2	Unspecified	Increased eutopic endometrial MMP-2 expression in EM and significantly lower TIMP-2
Uzan et al. (33)	MMP-2,−3, -11 TIMP-1,−2	Colorectal, endometrioma, peritoneal	MMP profile depends on EM location; Highest MMP-2,−3, and−11 expression in colorectal EM; TIMP-2 expression higher in peritoneal EM
Hudelist et al. (40)	MMP-1	Endometrioma, peritoneal	Significantly increased MMP-immunohistochemistry staining in ectopic lesions; non-significant differences in eutopic MMP-1 compared to controls
Kyama et al. (11)	MMP-3	Unspecified	Significantly higher eutopic MMP-3 mRNA expression in EM
Matsuzaki et al. (41)	MMP-9	Unspecified	Higher eutopic MMP-9 in EM; PFK 115-584 inhibited activity and reduced invasive cells
Cominelli et al. (32)	MMP-27	Endometrioma, peritoneal, rectovaginal	Increased macrophage MMP-27 in endometrioma and peritoneal EM, but not in rectovaginal lesions
Tang et al. (38)	MMP-2, -9 TIMP-1,−2, -3	Unspecified	Decreased MMP-2 DNA methylation in EM cells; Significantly higher MMP-2,−9 and TIMP-1,−2 transcription abundance in EM; Significantly lower TIMP-3 transcription abundance in EM

While many of the studies recognize that MMP is upregulated during menstruation, most of the evidence available is from tissue samples collected during the proliferative or secretory (31, 33, 35, 38) phases. This limits the interpretation that MMP dysregulation during menstruation can cause endometriosis and future research should compare endometrial tissue obtained during the menstrual phase to fully understand the impact on disease pathogenesis. Since the location can also affect MMP expression, endometriotic samples should be compared according to lesion location to minimize potential confounders.

## Hypoxia and Angiogenesis

During menstruation, the rapid decline in progesterone also causes vasoconstriction of the spiral arterioles which supplies oxygenated blood to the endometrium during the luteal phase. Reduced oxygen supply induces hypoxic stress stabilizing hypoxia inducible factor 1 (HIF-1). Although the exact roles of hypoxia and HIF-1 in the endometrium remains unclear, it is hypothesized to help restore endometrial blood supply and assist in endometrial repair following menstruation (42, 43). However, perturbation of hypoxia in the endometrium may also potentiate gynecological conditions like heavy menstrual bleeding and endometriosis (42).

One of the ways hypoxia may promote endometriosis is by increasing EMT. The EMT theory describes the changes of stationary epithelial cells to migratory mesenchymal cells during tissue repair. Our understanding of EMT in endometrial physiology is mostly derived from murine mice models, but the few studies utilizing human endometrial tissue suggest that factors driving EMT may be increased in endometriosis (44, 45). Rytkonen et al. found that hypoxia upregulated several stromal cell-specific genes that drive EMT (e.g., collagens, fibronectin, and proteases) and increased the expression of transcription factors JunD Proto-Oncogene and CCAAT Enhancer Binding Protein Delta by 18- and 5-fold, respectively, in deep endometriotic lesions (45). These transcription factors could be potential treatment targets, and inhibitors of the Jun pathway like the c-Jun NH_2_-terminal kinase inhibitor significantly reduced the amount of active endometriotic lesions in baboon models (46). Other studies also found increased mesenchymal transition markers like N-cadherin and vimentin in endometrial epithelial cells under hypoxic and inflammatory conditions (47, 48). Meanwhile, invasive activity and mesenchymal changes of Ishikawa cells decreased when HIF-1a levels were down-regulated (48). Therefore, hypoxic stress during menstruation may promote EMT and gene expression favoring endometrial cell migration.

Vascular endothelial growth factor alpha (VEGF-a) is an important angiogenic mediator, that is upregulated during menstruation and further activated by hypoxia and inflammation (49). However, excess VEGF-a in menstrual fragments may increase the vascularization potential of shed cells during retrograde menstruation facilitating attachment and growth at extra-uterine sites (9, 50). An interesting point of discussion is the comparison of VEGF expression in red (more active and vascular) lesions vs. black (less active and later-staged) lesions. Khan et al. found significantly higher VEGF expression in red lesions correlating with higher vascular activity (51). Meanwhile, a later study by Takehara et al. found no significant difference in gene expression and it remains unclear whether VEGF differs depending on the type of lesion (52). However, both agreed that women with endometriosis had significantly higher VEGF immunoreactivity in eutopic/ectopic endometrial tissue compared to controls which is well-supported by the existing literature (52–55). The endometrial lesions also exhibited similar proliferative and angiogenic activity as eutopic tissue supporting Sampson's theory of retrograde menstruation (51).

The Notch-induced four jointed box 1 (FJX1) protein has also been considered a possible pro-angiogenic factor in endometriosis. In the primate endometrium, Notch regulates decidualization, cell proliferation, and cell fate (56). Although FJX1 function is poorly understood in humans, its regulatory actions on HIF-1 may influence angiogenic activity. In eutopic tissue from human and baboon models with endometriosis, FJX1 was significantly increased during the secretory phase. During menstruation, FJX1's downstream effects like increased angiogenic activity and HIF-1 expression was observed (57). However, other upstream factors must be dysregulated in endometriosis since FJX1 was not significantly raised in the normal endometrium. Increased activation of the Notch signaling pathway in endometriosis could be a reasonable assumption since it induces FJX1 expression (58). In murine models with endometriosis, administration of Notch1 antagonists reduced cell migration and size of lesions and could be a promising therapeutic target (58).

While VEGF activity may be similar between eutopic and ectopic endometrial tissue, other menstrual characteristics may not necessarily be shared. In a recent retrospective study, matched superficial peritoneal endometriotic lesions and eutopic endometrial tissue from 42 patients were compared for histological/morphological analysis throughout the menstrual cycle and only 4% of the endometriotic lesions displayed stromal decidualization during the secretory phase (59). Endometriotic gland profiles (i.e., presence of hemosiderin-laden macrophages outside the menstrual phase) were independent of the menstrual cycle phases also reported by previous studies which may explain intermenstrual pelvic pain symptoms reiterating the complexity of endometriotic tissue (59–61).

## Control of Apoptosis Regulators and Cell Proliferation

Apoptosis is a form of programmed cell death and is important during menstruation to eliminate shed cells within the uterine environment. In women with endometriosis, reduced spontaneous eutopic endometrial apoptosis was observed using TdT-mediated dUTP biotin nick end-labeling assay throughout the menstrual cycle which could prolong cell survival for implantation at ectopic sites (62, 63). Interestingly, when Bax, a pro-apoptotic gene, was analyzed using immunohistochemical techniques during the secretory phase, the levels were raised in endometriosis which seems counterintuitive (63, 64). When another study further separated the secretory phase into early and late stages, Bax mRNA expression was significantly higher during the early phase in endometriosis, but decreased by 63% during the late secretory phase accompanied by reduced stromal and epithelial apoptotic activity compared to controls (64). It is unclear why pro-apoptotic factors are upregulated earlier in the menstrual cycle, but the later downregulation supports the theory of reduced apoptosis during menstruation in endometriosis.

Increased expression of anti-apoptotic factors may also explain the reduced apoptotic activity in endometriosis. For example, the phosphorylated ERK1/2 pathway usually prolongs cell survival and can influence the c-Jun transcription factor described earlier but is abnormally high in endometriosis regardless of the menstrual cycle phase resulting in persistent proliferative changes (65–68). Recently, a study suggested that the ERK pathways may also induce plasminogen activator inhibitor-1 expression, another anti-apoptotic protein that inhibits fibrinolysis, and is hypothesized to assist in the shedding of endometrial cells for attachment elsewhere (69).

The B-cell lymphoma 2 family proteins have also been commonly studied since they comprise of both pro-apoptotic (Bcl-xS) and anti-apoptotic (Bcl-xL and Bcl-2) regulators in the endometrium. In every sample analyzed, Bcl-xL expression significantly exceeded Bcl-xS throughout the menstrual cycle. Furthermore, the anti-apoptotic Bcl-2 form was increased in endometriosis improving endometrial cell survival (70). Overall, the literature suggests that the decreased apoptotic activity during the late-secretory and menstrual phases prolong the viability of shed endometrial cells allowing for implantation. However, the proliferative and early-secretory phases may have increased apoptotic activity and should be further investigated.

## Conclusions

**Figure 1:**
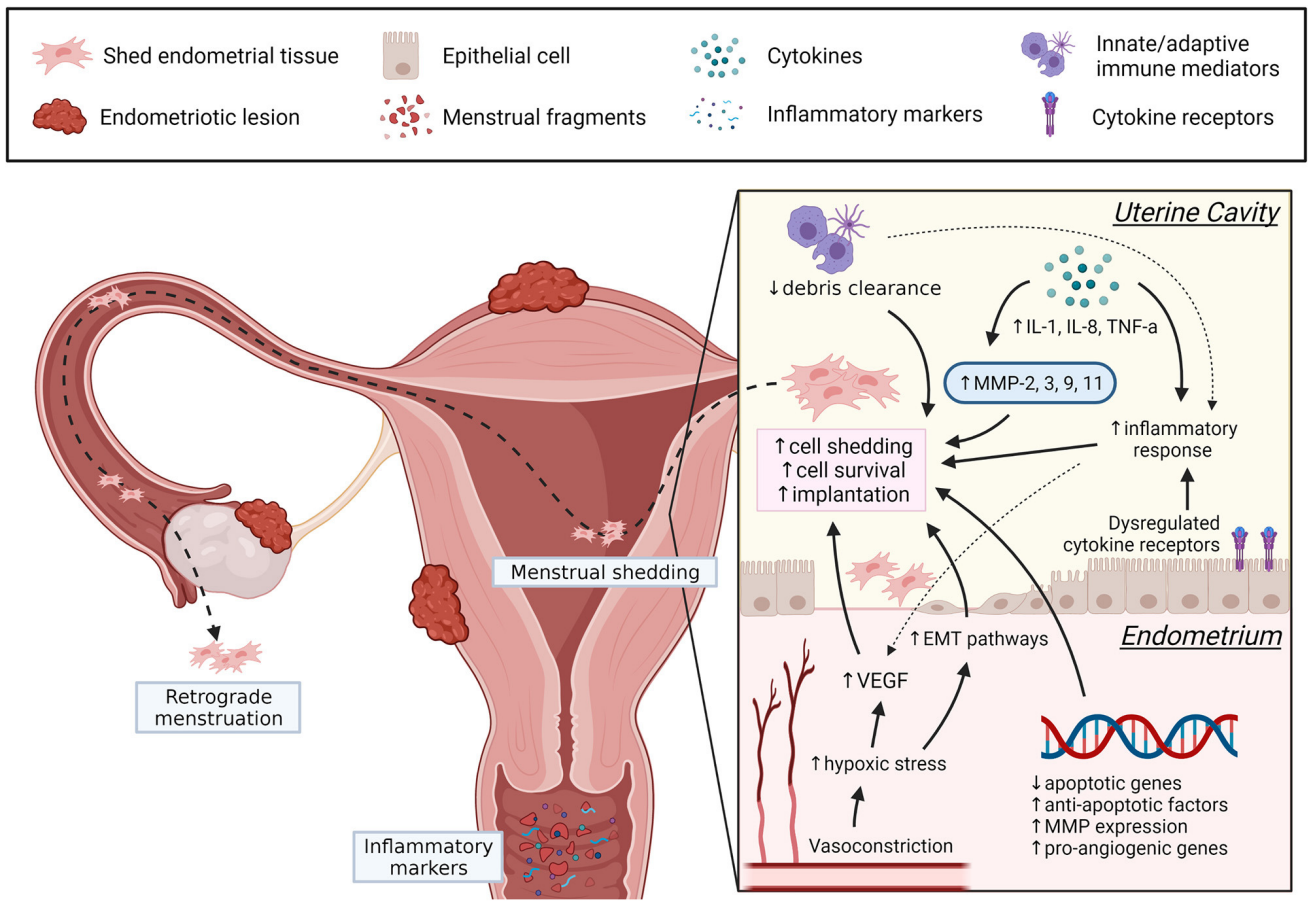
Summary of dysregulated menstrual pathways that can contribute to the pathogenesis of endometriosis. In women with endometriosis, dysregulation of several menstrual processes (angiogenesis, EMT, apoptotic regulators, inflammation, debris clearance, MMP) may promote endometrial cell detachment, prolong endometrial cell survival, and increase the likelihood for implantation following retrograde menstruation. Understanding these pathways may introduce new therapeutic targets and can be achieved by further exploring the underlying mechanisms of menstrual dysregulation in endometriosis. Created with BioRender.com.

Menstruation is a complex physiological process, and dysregulation of control mechanisms are implicated in abnormal uterine bleeding and endometrial diseases. In endometriosis, increased endometrial invasion, inflammation, angiogenic activity, and MMP have many overlapping pathways facilitating basalis invasion, EMT, stem cells release, prolonged viability of shed endometrial cells and implantation of ectopic lesions (see Figure 1), although the exact underlying mechanisms remain unclear. In the future, more studies should assess endometrial biopsies collected during the menstrual phase. The menstrual cycle phase of biopsy collection and the location of endometriosis lesions may introduce confounders when analyzing regulatory factors and should be considered. Evaluating menstrual blood from women with endometriosis could also be a useful non-invasive sample for understanding disease mechanisms and exploring potential biomarkers. It is evident that menstruation is a complex physiological process with many unanswered questions. However, understanding how dysregulation of certain factors contribute to the pathogenesis of endometriosis can help identify new diagnostic markers and therapeutic targets, ultimately improving the patient's quality of life.

## Author Contributions

KK wrote the manuscript, created the tables/figures, and designed the review with AH. DG, LW, and AH were involved in critically reviewing and editing the manuscript. All authors contributed to the article and approved the submitted version.

## Funding

AH and LW were supported by a grant from the Medical Research Council (MR/N022556/1). DG was supported by a Sir Henry Dale Fellowship jointly funded by the Wellcome Trust and the Royal Society (Grant No. 220656/Z/20/Z to DG). LW was supported by an NES/CSO Clinical Lectureship (PCL/19/01).

## Conflict of Interest

LW receives grant funding from the National Institute for Health Research NIHR and the Chief Scientist's Office. AH receives grant funding from the NIHR, the Medical Research Council MRC, the Chief Scientist's Office, and Roche. He has received honoraria for consultancy for Ferring, Roche, Nordic Pharma, and Abbvie. The remaining authors declare that the research was conducted in the absence of any commercial or financial relationships that could be construed as a potential conflict of interest.

## Publisher's Note

All claims expressed in this article are solely those of the authors and do not necessarily represent those of their affiliated organizations, or those of the publisher, the editors and the reviewers. Any product that may be evaluated in this article, or claim that may be made by its manufacturer, is not guaranteed or endorsed by the publisher.
